# MEFV mutations in Iranian children with systemic onset juvenile idiopathic arthritis

**DOI:** 10.1186/1546-0096-12-S1-P226

**Published:** 2014-09-17

**Authors:** Shirin Farivar, Mahdieh Hasani, Reza Shiari

**Affiliations:** 1 Genetics, Shahid Beheshti University, Tehran, Iran, Islamic Republic Of; 2 Pediatric Rheumatologist, Mofid Children’s Hospital, Tehran, Iran, Islamic Republic Of

## Introduction

Systemic onset Juvenile Idiopathic Arthritis (SoJIA) is a subset of juvenile idiopathic arthritis (JIA) that describes patients with intermittent fever, arthritis and skin rash with complex genetic trait in children less than 16 years old. The etiology of disease has not yet been identified; but because of high level of IL-1β in the blood of patients; and relation between its complications, and clinical signs of disease; the only theory is mutation in factors which are involved in the control of production and secretion of this proinflammatory cytokine, like IL-1Ra, IL-1 type II decoy receptor proteins and soluble IL-1R accessory protein (IRAP).

## Objectives

The aim of this study was to identify the association between MEFV mutations in exons 2 and 10, and SoJIA disease in 30 Iranian patients.

## Methods

Thirty Iranian children with SoJIA disease and 30 healthy control, were screened for MEFV mutations in exons 2 and 10 by sequencing. All patients were diagnosed according to the classification criteria of EULAR for SoJIA. The control group consisted of 30 healthy individuals (15 female and 15 male) who did not have any history of immune system disorders and other diseases with known genetic or hereditary predisposition.

## Results

Statistical analysis showed that the MEFV mutations in the patients and control groups were significantly different (p<0.01). The mutations were detected in 17 of 30 patients (56.6%). The respective figure for the control subjects was 1 of 30 (3.3%). Leading mutation was R202Q mutation which detected in 13 patients ( 6 homozygote and 7 heterozygote). Following eight patients had M694V mutation ( 2 homozygote and 6 heterozygote). Three patients had E148Q mutation in heterozygote form.

## Conclusion

MEFV mutations may be an important cause of high level of IL-1β in SoJIA patients. It shows that SoJIA is better to be classified as auto-inflammatory disorder.

## Disclosure of interest

None declared.

